# Analysis of multivariate longitudinal immuno-epidemiological data using a pairwise joint modelling approach

**DOI:** 10.1186/s12865-021-00453-5

**Published:** 2021-09-17

**Authors:** Lawrence Lubyayi, Patrice A. Mawa, Stephen Cose, Alison M. Elliott, Jonathan Levin, Emily L. Webb

**Affiliations:** 1 Department of Epidemiology and Biostatistics, School of Public Health, University of the Witwatersrand, Johannesburg, South Africa; 2 Immunomodulation and Vaccines Programme, Medical Research Council/Uganda Virus Research Institute and London School of Hygiene and Tropical Medicine Uganda Research Unit, Plot 51-59 Nakiwogo Road, P.O. Box 49, Entebbe, Uganda; 3 Uganda Virus Research Institute, Entebbe, Uganda; 4 Department of Infection Biology, London School of Hygiene and Tropical Medicine, London, UK; 5 Department of Clinical Research, London School of Hygiene and Tropical Medicine, London, UK; 6 MRC International Statistics and Epidemiology Group, Department of Infectious Disease Epidemiology, London School of Hygiene and Tropical Medicine, London, UK

**Keywords:** Latent *Mycobacterium tuberculosis* infection, BCG vaccine, Cytokine responses, Linear mixed model, Pairwise joint modelling

## Abstract

**Background:**

Immuno-epidemiologists are often faced with multivariate outcomes, measured repeatedly over time. Such data are characterised by complex inter- and intra-outcome relationships which must be accounted for during analysis. Scientific questions of interest might include determining the effect of a treatment on the evolution of all outcomes together, or grouping outcomes that change in the same way. Modelling the different outcomes separately may not be appropriate because it ignores the underlying relationships between outcomes. In such situations, a joint modelling strategy is necessary. This paper describes a pairwise joint modelling approach and discusses its benefits over more simple statistical analysis approaches, with application to data from a study of the response to BCG vaccination in the first year of life, conducted in Entebbe, Uganda.

**Methods:**

The study aimed to determine the effect of maternal latent *Mycobacterium tuberculosis* infection (LTBI) on infant immune response (TNF, IFN-γ, IL-13, IL-10, IL-5, IL-17A and IL-2 responses to PPD), following immunisation with BCG. A simple analysis ignoring the correlation structure of multivariate longitudinal data is first shown. Univariate linear mixed models are then used to describe longitudinal profiles of each outcome, and are then combined into a multivariate mixed model, specifying a joint distribution for the random effects to account for correlations between the multiple outcomes. A pairwise joint modelling approach, where all possible pairs of bivariate mixed models are fitted, is then used to obtain parameter estimates.

**Results:**

Univariate and pairwise longitudinal analysis approaches are consistent in finding that LTBI had no impact on the evolution of cytokine responses to PPD. Estimates from the pairwise joint modelling approach were more precise. Major advantages of the pairwise approach include the opportunity to test for the effect of LTBI on the joint evolution of all, or groups of, outcomes and the ability to estimate association structures of the outcomes.

**Conclusions:**

The pairwise joint modelling approach reduces the complexity of analysis of high-dimensional multivariate repeated measures, allows for proper accounting for association structures and can improve our understanding and interpretation of longitudinal immuno-epidemiological data.

**Supplementary Information:**

The online version contains supplementary material available at 10.1186/s12865-021-00453-5.

## Background

Longitudinal studies are indispensable for the investigation of changes in outcomes over time. Through making measurements on study participants over time, longitudinal studies allow the direct study of temporal changes within individuals and the factors that influence these changes [1]. Since the study of change is fundamental to almost every discipline, there has been a steady growth in the number of studies using longitudinal designs [1]. However, many challenges arise when analysing data from longitudinal studies [2]. Naturally, the repeated measures arising from longitudinal studies are multi-dimensional and have a complex random-error structure that must be appropriately accounted for in the analysis [1]. Problems of missing data and attrition are also common in these studies; yet appropriate handling of missing data continues to pose one of the greatest challenges in their analysis [1, 3, 4]. These and many other issues increase the complexity of longitudinal data analysis, and this is particularly the case for immuno-epidemiological studies. Immuno-epidemiological studies investigate the influence of population immunity on the epidemiology of conditions such as infectious diseases, cancer, hypersensitivity and autoimmunity [5, 6]. Such studies are likely to have a large number of, often correlated, outcomes measured repeatedly over time.

Immuno-epidemiological studies have increased tremendously in the recent past [7]. However, the complexity of relationships between multitudes of immuno-epidemiological parameters poses the challenge of selection of the most suitable statistical methods for extraction of the greatest amount of pertinent information from such complex datasets [7]. In many immuno-epidemiological studies, simple statistical approaches are applied even when complex patterns of inter-relationships between parameters are expected [7, 8]. A number of articles have given an overview of application of statistical techniques to immuno-epidemiological data [7–10], however these focus mainly on cross-sectional data. To our knowledge, only one paper [11] has aimed to provide guidance on the analysis of longitudinal immunological data, and none have focused on longitudinal immunological data with multivariate outcomes.

In immuno-epidemiological studies, a number of scientific questions might be of interest: for instance, to determine the effect of an intervention or exposure on the joint evolution of all outcomes together, or to study the association between evolutions of different outcomes. Modelling the different outcomes separately may not be appropriate because it ignores the underlying relationships between them. In such situations, a joint modelling strategy is necessary [12]. This paper describes methods that can be applied in this context, with emphasis on the pairwise joint modelling approach and its benefits over more simple statistical analysis approaches. The pairwise joint modelling approach has been previously applied to multivariate longitudinal lipid profiles data from a heart study [13], high-dimensional longitudinal profiles of hearing thresholds [14], and to longitudinal multivariate markers of renal graft failure [15]. Approaches are demonstrated using data from the Infant BCG Study (IBS) [16] which was carried out in Entebbe, Uganda. The primary aim of the study was to determine the effect of prenatal exposure to maternal LTBI on the infant immune response (cytokine responses (IL-2, IL-5, IL-10, IL-13, IL-17A, TNF, and IFN-γ) to the *M.tb* purified protein derivative (PPD)) following immunisation with BCG [16]. Additional questions of interest were (i) the strength of association between the evolutions of cytokine responses over time, (ii) the effect of LTBI on the joint evolution of cytokine responses over time, and (iii) whether the relationship between cytokine responses differed comparing pre- and post-BCG time points. All these questions necessitate a joint modelling strategy. Findings from these objectives will inform BCG vaccination policy, specifically addressing whether BCG vaccination at birth is likely to be beneficial to all infants, irrespective of maternal LTBI status.

## Methods

### Study design, participants and data

The IBS has been described previously [16]. Briefly, the IBS was an observational longitudinal immuno-epidemiological study. The study successfully enrolled infants born to mothers with (n = 132) or without (n = 150) LTBI and followed them up for one year. Since the study fell short of the targeted 150 respondents for mothers with LTBI, there was a slight reduction in power from 80 to 78% for the specified scenario. Blood samples were taken at selected time points throughout infancy, with more frequent sampling in early infancy when immune responses have been shown to change most rapidly [17]. There was random assignment of individual infants to two sampling strategies, in a 1:1 ratio, to reduce the blood-sampling burden. The first sampling strategy comprised collection of 2 ml venous blood at 1, 6, and 14 weeks, and 5 ml venous blood at 52 weeks; the second sampling strategy comprised collection of 2 ml venous blood at 4, 10, and 24 weeks, and 5 ml venous blood at 52 weeks. All infants were BCG immunised at birth or within the first week of life with BCG (Statens Serum Institut (SSI), Denmark). Immunological parameters measured included 7 cytokine responses (IL-2, IL-5, IL-10, IL-13, IL-17A, TNF, and IFN-γ) to PPD. These were assessed by Luminex (Bio-Rad Luminex® 200 system and Bioplex Manager Software version 6.1 (Bio-Rad)) in 6-day whole blood cultures, in cord blood and at weeks 1, 4, 6, 10, 14, 24 and 52. Net cytokine response values were log-transformed to meet distributional assumptions while fitting mixed effects models.

### Conceptual framework

**Fig. 1:**
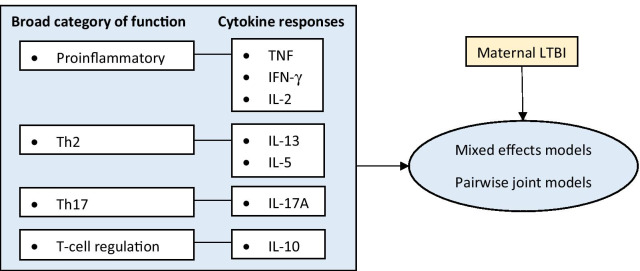
Conceptual framework

Figure 1 highlights the multivariate longitudinal outcomes, cytokine responses (IL-2, IL-5, IL-10, IL-13, IL-17A, TNF, and IFN-γ) to PPD. It shows the underlying immunological functions for the different cytokines namely proinflammatory, T helper (Th)2, Th17 and T-cell regulation. The figure also shows that mixed effects and pairwise joint models were applied to study the evolution of cytokine responses over time and how that evolution depends on maternal LTBI status.

### Statistical software

Data analysis was conducted using SAS version 9.4 (SAS Institute, Cary NC, USA), Stata 15.0 (College Station, Texas, USA) and R version 3.6.0 (R Foundation for Statistical Computing, Vienna, Austria). A 5% significance level was used for all analyses.

### Participants’ baseline characteristics

Baseline characteristics of participants were summarised, by LTBI status, using percentages, means and standard deviations, and medians and interquartile ranges.

### Simple analyses ignoring correlations between time points and outcomes

Simple analyses often reported for such cytokine response data include t-tests if the distributions are approximately normal and non-parametric alternatives such as Mann–Whitney tests when the distributional assumptions are relaxed. Such tests are usually conducted separately for individual cytokine responses and separately at each time point. Adopting this simple approach, our initial analyses employed Mann–Whitney tests comparing responses between infants born of mothers with or without LTBI. Conservative Bonferroni corrections were applied to demonstrate their use in adjusting for multiple comparisons at each time point.

### Graphical exploration of longitudinal profiles

Individual profile plots were constructed for each of the seven longitudinal outcomes to obtain insight into how infant responses evolved over time as well as to give an indication of between and within infant variability. When this kind of variability is present it provides the motivation for modelling approaches which take this into consideration, most commonly via the specification of random effects.

Average profile plots were then constructed, for each outcome, to describe the mean evolution of infant responses, disaggregated by maternal LTBI status. These plots give an indication of the functional form of the evolution and an initial idea of whether this evolution differs by maternal LTBI status.

### Analyses allowing for longitudinal data but ignoring correlations between different outcomes

Changes in log-transformed cytokine responses over time, by mother’s LTBI status, were studied using univariate linear mixed models (LMM) adjusted for factors that showed baseline differences between the two groups.

The use of the linear mixed model for analysing univariate longitudinal data has been discussed extensively [3, 18–21]. The model handles continuous longitudinal data in an easy, valid and flexible manner [22] and can be used for data with an unequal number of measurements per subject [18]. The model is defined as

$${\varvec{Y}}_{i} = {\varvec{X}}_{i} {\varvec{\beta}} + {\varvec{Z}}_{i} {\varvec{b}}_{i} + {\varvec{\varepsilon}}_{i} ;\quad {\varvec{b}}_{i} \sim N\left( {0, D} \right); {\varvec{\varepsilon}}_{i} \sim N\left( {0, {\varvec{\varSigma}}_{i} } \right);\quad {\varvec{b}}_{i} , {\varvec{\varepsilon}}_{i} \,are\, independent$$

where ***Y***_*i*_ is the *n*_*i*_ dimensional response vector for the *i*th subject, 1 ≤ *i* ≤ *N*, *N* is the number of subjects, ***X***_*i*_ and ***Z***_*i*_ are (*n*_*i*_ × *p*) and (*n*_*i*_ × *q*) dimensional matrices of known covariates, ***β*** is a *p*-dimensional vector of fixed effects, ***b***_*i*_ is a *q*-dimensional vector of random effects, ***ε***_*i*_ is an *n*_*i*_-dimensional vector of residual components, *D* is a covariance matrix of random effects and ***Σ***_*i*_ is a covariance matrix of residuals [18]. Random effects represent an aggregation of all unobserved or unmeasured factors that make individuals respond differently to each other [21].

Longitudinal immuno-epidemiological data are often characterised by non-linear patterns over time. Such patterns can still be handled under the linear mixed effects models framework with power transformations of time. Fractional polynomials (FPs) are characterised by power terms which can be negative values and/or fractions. Conventional polynomials (CPs) are a special case of FPs with power terms having only integer values. FPs have been shown to have more favourable characteristics than higher order CPs when modelling non-linear growth curves within the context of the linear mixed model [23–25]. The R package ‘mfp’ [26] was used to determine the best fitting FP for each outcome. Graphics and criteria such as the Akaike Information Criteria (AIC), Deviance, R^2^ and adjusted R^2^ were used to compare the best fitting FP to the best higher order CP (which had been chosen using graphical means).

Under the LMM framework, likelihood based tests, specifically Restricted Maximum Likelihood (REML), were used to check the need for inclusion of various serial correlation structures (simple, autoregressive, compound symmetry, unstructured, exponential, power and gaussian). Mixtures of chi-square distributions were then used to assess the need for extending the random effects structures. Finally, to discover the most parsimonious mean structure, likelihood ratio tests were employed under maximum likelihood estimation.

### Analyses allowing for longitudinal data and correlations between outcomes

Patterns of correlation between the different outcomes were expected, for instance, certain cytokines are associated with particular cell types or functions (such as T-helper (Th)1, Th2 or regulatory functions). A statistical modelling approach which accounts for such correlation structures was necessary, to provide additional insight into the data. The seven univariate linear mixed models were thus combined into a full multivariate model resulting in a 7 × 7 covariance matrix for random effects (if only random intercepts are considered) and a 7 × 7 covariance matrix for error components. Each of these two matrices had 28 variance–covariance components (i.e. 7 variance components + 21 covariance components). This then resulted in a total of 28*2 = 56 covariance parameters altogether. This dimensionality of random effects is too large for standard software packages to handle.

The pairwise joint modelling approach was introduced as a novel procedure for fitting such random effects models without restricting the dimensionality [14]. The general idea is that all parameters in the full multivariate model can be identified from fitting all bivariate models for each pair of outcomes separately, then, afterwards, estimates are averaged to obtain one single estimate for each parameter of the full joint model. For standard errors, in order to correctly calculate the sampling variability of the estimates from the pairwise approach, pseudo-log-likelihood estimation is applied [14, 27]. This approach was applied to our data in order to properly account for and to study the strength of association between the evolutions of cytokine responses over time. Parameter estimates from this approach were used to construct a Wald-type test statistic for the joint effect of maternal LTBI on evolution of all outcomes together. Principal components analysis (PCA) was then carried out on the 7 × 7 correlation matrix of random effects and the results were compared to PCA of cord blood cytokine responses to establish whether the clustering of responses before immunisation with BCG differed from the clustering of responses after BCG. A SAS macro [28] was adapted and used to implement the pairwise joint modelling approach.

## Results

### Participants’ baseline characteristics

Between June 2014 and October 2016, the IBS enrolled infants born to mothers with (n = 132) or without (n = 150) LTBI and followed them up for one year. Baseline characteristics of participants are shown in Table 1 by LTBI status. LTBI-positive mothers were on average older, more likely to have lived with someone who had TB, to drink alcohol and to originate from the central region of Uganda. Infant characteristics (sex and birth weight) and other maternal characteristics were similar between the two groups.

**Table 1:** Baseline characteristics of study participants

	LTBI negative (n = 150)		LTBI positive (n = 132)	
*Maternal characteristics*				
Mean mother's age (SD) (mv 0, 3)^a^	23.65	(3.67)	25.53	(4.99)
Median number of pregnancies (IQR) (mv 1, 1)	2	(1–3)	2	(2–4)
Positive malaria test during pregnancy (mv 1, 1)	38	25.5%	29	22.1%
Ever lived with someone with TB (mv 2, 1)	3	2.0%	19	14.5%
BCG scarring (mv 1, 2)	106	71.1%	95	73.1%
Current marital status (mv 2, 4)				
Single	28	18.9%	20	15.6%
Married/living as married	120	81.1%	108	84.4%
Drink alcohol (mv 3, 4)	18	12.2%	28	21.9%
Mother's tribe grouping (mv 5, 3)				
Central	56	38.6%	72	55.8%
Other	89	61.4%	57	44.2%
Father's tribe grouping (mv 3, 2)				
Central	58	39.5%	75	57.7%
Other	89	60.5%	55	42.3%
*Infant characteristics*				
Sex of the baby, male	77	51.3%	77	58.3%
Mean birth weight in kg (SD)	3.24	(0.43)	3.21	(0.40)

Data are mean (SD), median (IQR), or n (%)

*SD* standard deviation, *IQR* interquartile range, *mv* missing values

^a^Figures in parentheses indicate missing values in the LTBI-Negative and LTBI-Positive groups, respectively

Due to the study design, not all infants provided samples at all time points, sample numbers assayed at each time point are shown (Additional file 1: Table S1).

### Simple analyses ignoring correlations between time points and outcomes

Table 2 shows unadjusted p-values from the Mann–Whitney test for the comparisons of cytokine responses between infants born to mothers with or without LTBI at each time point for all the seven outcomes. Statistically significant differences between the two groups are highlighted at week 4 (for cytokines IFN-γ, IL-17A, and IL-10), week 10 (for TNF) and at week 24 (for IL-17A). When a Bonferroni correction is applied at each of these time points, none of the differences remains significant.

**Table 2:** P-values from Mann–Whitney tests for the comparison of cytokine responses to PPD between the two infant groups

Outcome	Cord blood	Week1	Week4	Week6	Week10	Week14	Week24	Week52
TNF	0.060	0.697	0.137	0.096	**0.032**	0.589	0.253	0.377
IFN-γ	0.891	0.691	**0.026**	0.203	0.703	0.299	0.055	0.121
IL-2	0.982	0.643	0.400	0.948	0.629	0.601	0.743	0.071
IL-5	0.729	0.430	0.864	0.750	0.147	0.472	0.156	0.688
IL-13	0.888	0.915	0.351	0.715	0.178	0.620	0.496	0.375
IL-17A	0.353	0.852	**0.047**	0.408	0.144	0.418	**0.040**	0.841
IL-10	0.056	0.959	**0.014**	0.187	0.171	0.293	0.765	0.538

Unadjusted *p*-values for the comparisons of responses between infants born of LTBI positive or negative mothers at each time point. Values in bold are significant at the 5% level (without considering the Bonferroni correction for multiple testing)

### Graphical exploration of longitudinal profiles

**Fig. 2:**
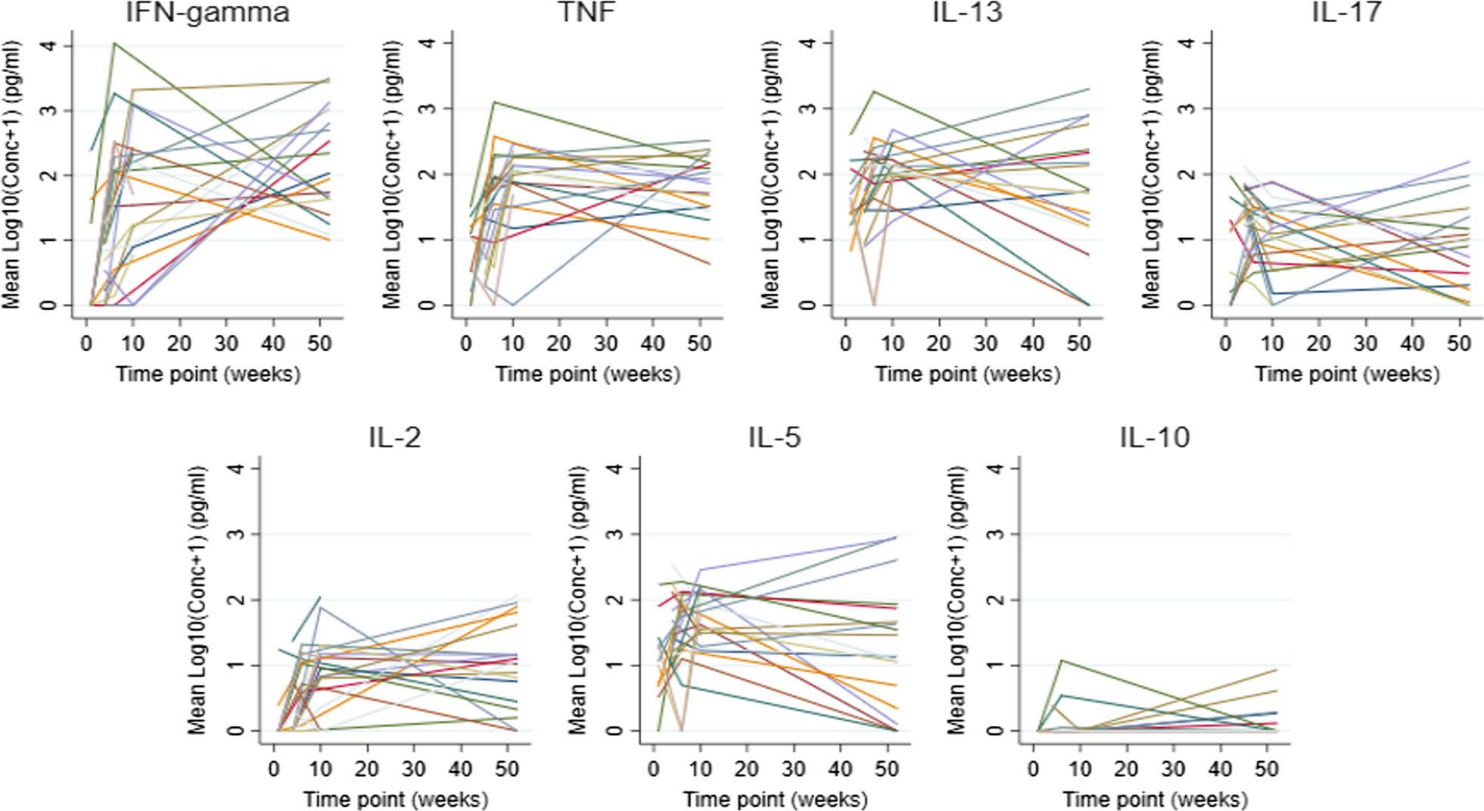
Individual profiles for a sample of 30 infants. PPD-specific cytokine responses (on the log scale) for a consecutive sample of 30 infants, for each of the 7 cytokines considered. Each infant is represented by a single line

Figure 2 shows the individual profiles for a consecutive sample of 30 infants, for each of the seven cytokine responses to PPD. It is evident from the figure that the infants have highly variable concentrations at the start, this suggests that perhaps linear mixed models with random intercepts could be an appropriate modelling approach.

**Fig. 3:**
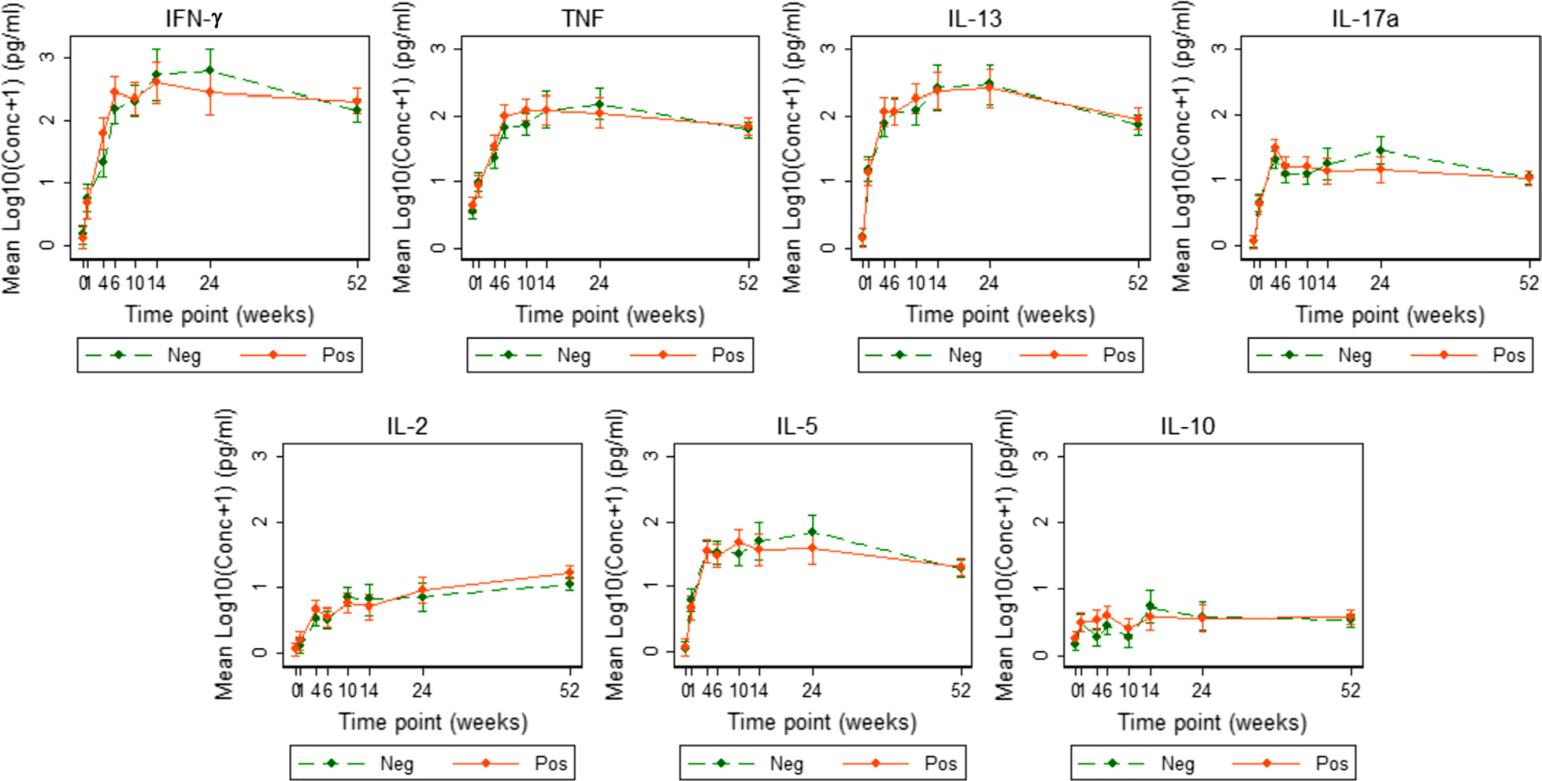
Average evolutions of cytokine responses to PPD in infants of mothers with or without LTBI. Average PPD-specific cytokine concentrations corrected for background (on the log scale). Points represent mean values and the bars represent 95% confidence intervals for the mean. The solid and dashed lines represent concentrations from children born of LTBI-positive and LTBI-negative mothers respectively

Figure 3 shows the average evolutions of the seven cytokine responses over time, stratified by mothers’ LTBI status. It can be seen that in general there was a sharp increase up to about 10 weeks and then a plateauing through to 52 weeks, with some curvature which should be appropriately considered during model building. These patterns of evolution point to the consideration of fractional polynomials for modelling the mean structure of the outcomes. It is also seen that cytokine responses to PPD were similar, at all time points, between the two infant groups.

### Analyses allowing for longitudinal data but ignoring correlations between different outcomes

First or second order FPs, providing the best fit for each of the seven outcomes, were identified using the R function ‘mfp’. Both TNF and IFN-γ responses were best represented by second order FPs with powers m1 = 0.5 and m2 = 0.5, IL-13 with powers m1 =  −0.5 and m2 = 3, IL-17A with powers m1 =  −2 and m2 =  −2, IL-5 with powers m1 =  −1 and m2 = 3. IL-2 was best represented by a first order FP with m1 = 0 (equivalent to a log transformation of time) while IL-10 was best represented by a linear function of time. For all the seven outcomes, FP models had better fit criteria (lower AIC and Deviance and higher R^2^) than conventional higher order polynomials (Additional file 1: Table S2). Graphical comparisons of FPs and higher order CPs show better performance for the FP models especially at the extreme ends (Additional file 1: Figures S1A and S1B).

**Fig. 4:**
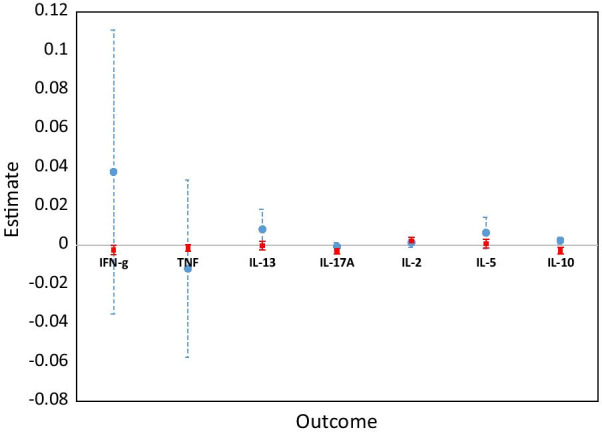
Estimates for interaction effects of time and LTBI status from univariate and pairwise models. Estimates and 95% confidence intervals for the interaction effects of time and mother’s LTBI status from univariate linear mixed models (solid circles and associated dashed whiskers) and from the pairwise joint modelling approach (solid squares and whiskers)

Univariate linear mixed models were fitted for each outcome, using the identified FP functions to capture the curvature over time. Random intercepts were included in each of the seven models to account for the variability between different infants; extending the random effects structure to linear slopes of time was not necessary (based on the mixture of chi-square tests). Each model was adjusted for sex of the infant and for factors that showed baseline differences between the two groups (mother’s age, household TB contact, alcohol consumption, central region origin). There was no evidence of interaction between time and maternal LTBI status for any of the seven outcomes (Fig. 4).

### Analyses allowing for longitudinal data and correlations between outcomes

Table 3 shows the parameter estimates and standard errors for the interaction effects of time and mother’s LTBI status, for all the seven outcomes, obtained from the pairwise approach. A joint statistical test of the effect of LTBI on the evolutions of all outcomes together yielded a Wald-type test statistic with a chi-square value of 11.04, which was not significant when compared to the chi-square distribution with 7 degrees of freedom (p-value = 0.137), implying that LTBI had no effect on the evolution of all outcomes (when considered jointly).

**Table 3:** Estimates and standard errors from pairwise model

Outcome	Estimate	Std.Error
IFN-γ	− 0.00227	0.0025
TNF	− 0.00116	0.0016
IL-13	− 0.00002	0.0022
IL-17A	− 0.00265	0.0015
IL-2	0.00231	0.0019
IL-5	0.00102	0.0021
IL-10	− 0.00264	0.0017

Parameter estimates and standard errors for the interaction effects of time and mother’s LTBI status obtained from the pairwise joint modelling approach

Figure 4 also shows the interaction effects of time and mothers’ LTBI status, from the pairwise approach. The message is consistent with that from the univariate models, but with an added benefit of improved precision indicated by smaller 95% confidence intervals.

**Fig. 5:**
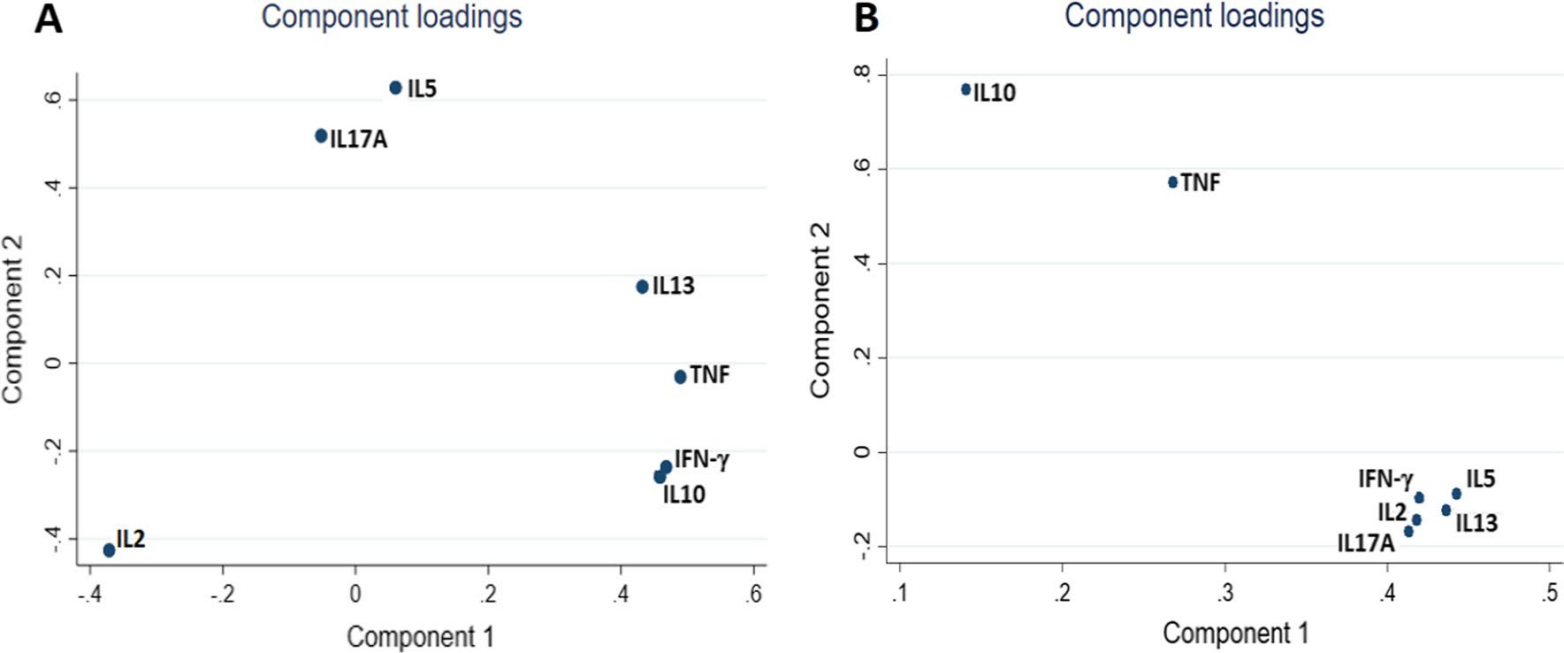
Principal components analysis of the correlation matrix of random intercepts and of cord blood outcomes. **a** Component loadings for the seven outcomes based on the 7 × 7 correlation matrix of random intercepts from the pairwise approach. **b** Component loadings for the seven outcomes based on cord blood responses to PPD

Figure 5a shows results of a PCA on the 7 × 7 correlation matrix of random intercepts (Additional file 1: Table S3). The result indicates that IL-5 and IL-17A responses evolved in a similar way, TNF, IFN-γ, IL-10 and IL-13 responses evolved similarly to each other, whereas IL-2 responses evolved uniquely from the other cytokines. Figure 5b shows the component loadings for the seven outcomes, based on cord blood responses. Comparison with Fig. 5a indicates that the association structure of the infant cytokine responses changed after the infants were immunised with BCG.

## Discussion

This paper describes a pairwise joint modelling approach and discusses its benefits over more simple statistical analysis approaches, commonly used for immuno-epidemiological data, with application to data from the Infant BCG Study. It demonstrates that certain scientific questions cannot be addressed by simple approaches. The pairwise approach is shown to be essential in situations where it is desired to determine the effect of a treatment on the joint evolution of all outcomes and when it is desired to study the relations between evolutions of longitudinal multivariate outcomes.

Simple statistical analysis approaches such as the Mann–Whitney test are often used for multivariate longitudinal immuno-epidemiological data [7, 8]. These ignore the correlation between repeated measurements over time and cannot be used to study the changes that happen between correlated outcomes over time. However, these simple approaches are quite often erroneously interpreted to show changes over time yet they ought to be interpreted as analyses at the separate time points.

Univariate linear mixed models are shown in this study. These provide an improvement over the simple approaches, for handling of longitudinal data. They handle continuous longitudinal data in an easy, valid and flexible manner [22], account for correlation between repeated measurements from individuals and can be valid depending on the nature of research question at hand. However, they analyse each outcome separately, ignoring the correlation between multiple outcomes assessed over the various time points.

The focus of this paper is on a more appropriate statistical analysis approach for multivariate longitudinal immuno-epidemiological data, that accounts for both the correlation between measurements from an individual over time and also the correlation between the multiple outcomes assessed at each time point. With this approach, the seven longitudinal outcomes from the IBS data were jointly modelled, considering a random intercept for each outcome. This led to a covariance structure with 56 parameters. Fitting a full multivariate model with maximum likelihood estimation was not possible when four or more of the seven outcomes were considered. This task was broken down into the fitting of 21 bivariate models via the pairwise approach as described [14, 27].

Our results show that the parameter estimates from the pairwise approach had better precision than those from the univariate mixed models. This could be attributed to the fact that the pairwise approach accounts for more variability (correlation of outcomes). It has been emphasized elsewhere, though, that gains in precision compared to univariate models should not be the key motivation for choosing the pairwise approach [27].

A major advantage of the pairwise approach is the opportunity to carry out a joint statistical test for the effect of LTBI on the evolution of all cytokine response outcomes together. This overcomes the multiple testing problem inherent with multiple outcome data measured at multiple time points, and can only be done under a full multivariate modelling approach, and not under any of the other simple or univariate approaches. This supported the conclusion that there was no difference between infants born of mothers with or without LTBI.

Another benefit of the pairwise approach, in our case, lies in the ability to estimate the association structures of the longitudinal outcomes and how these relate to each other. This can improve our understanding and interpretation of longitudinal immuno-epidemiological data. In our case, the PCA of cord blood responses suggested distinct groups with IL-10 separate from Th1 or Th2 cytokine responses. However, these initial patterns were not reflected in the profile of response that developed following immunisation with BCG as indicated by the PCA on the correlation matrix of random intercepts. Biologically, this suggests that the effect of BCG immunisation was potent enough to over-ride patterns established in *utero*, however, in the absence of a non-BCG control group this remains unconfirmed.

A limitation of our study could be in the potential influence of missing data. The mixed models used have an advantage of accommodating unbalanced data structures but they assume that data are missing at random. This assumption is inherently untestable however, and sensitivity analyses under alternative assumptions are recommended.

## Conclusion

The pairwise joint modelling approach for multivariate longitudinal data has utility for immuno-epidemiological data. It reduces the complexity of analysis of multivariate repeated measures of a relatively high dimension, while still accounting for association structures, thus providing an improvement over the simple univariate approaches in common use. The proposed approach can improve our understanding and interpretation of longitudinal immuno-epidemiological data.

## Supplementary Information

**Additional file 1.**: **Table S1** Number of available samples at each time point for responses to PPD. **Table S2** Comparison of Fractional polynomial and conventional higher order polynomials using various fit-statistics. **Table S3** Correlation matrix of random effects from the pairwise model. **Fig. S1A** Observed and fitted mean functions for the cytokine responses to PPD. **Fig. S1B** Observed and fitted mean functions for the cytokine responses to PPD.

## Data Availability

The data sets analysed in this study are available from the corresponding author on reasonable request.

## Abbreviations

BCG: Bacille Calmette–Guerine
IBS: Infant BCG Study
*M.tb*: *Mycobacterium tuberculosis*
LTBI: Latent *Mycobacterium tuberculosis* infection
TNF: Tumor Necrosis Factor
IFN: Interferon
IL: Interleukin
PPD: Purified protein derivative
Th: T-helper
FPs: Fractional polynomials
CPs: Conventional polynomials
LMM: Linear mixed model
AIC: Akaike Information Criteria
REML: Restricted Maximum Likelihood
PCA: Principal components analysis

## References

[1] Fitzmaurice G, Davidian M, Verbeke G, Molenberghs G: Advances in longitudinal data analysis: an historical perspective. In: Longitudinal data analysis. Chapman and Hall/CRC; 2008: 17–42.

[2] Caruana EJ, Roman M, Hernández-Sánchez J, Solli P (2015). Longitudinal studies. J Thorac Disease.

[3] Diggle PJ, Heagerty P, Liang K-Y, Heagerty PJ, Zeger S. Analysis of longitudinal data: Oxford University Press; 2002.

[4] Molenberghs G, Kenward M. Missing data in clinical studies, vol. 61: Wiley; 2007.

[5] Hellriegel B (2001). Immunoepidemiology–bridging the gap between immunology and epidemiology. Trends Parasitol.

[6] Krause PJ, Kavathas PB, Ruddle NH. Immunoepidemiology: Springer; 2019.

[7] Genser B, Cooper PJ, Yazdanbakhsh M, Barreto ML, Rodrigues LC (2007). A guide to modern statistical analysis of immunological data. BMC Immunol.

[8] Genser B, Fischer JE, Figueiredo CA, Alcântara-Neves N, Barreto ML, Cooper PJ, Amorim LD, Saemann MD, Weichhart T, Rodrigues LC (2016). Applied immuno-epidemiological research: an approach for integrating existing knowledge into the statistical analysis of multiple immune markers. BMC Immunol.

[9] McGuinness D, Bennett S, Riley E (1997). Statistical analysis of highly skewed immune response data. J Immunol Methods.

[10] Bennett S, Riley E (1992). The statistical analysis of data from immunoepidemiological studies. J Immunol Methods.

[11] Panda A, Chen S, Shaw AC, Allore HG (2013). Statistical approaches for analyzing immunologic data of repeated observations: a practical guide. J Immunol Methods.

[12] Verbeke G, Fieuws S, Molenberghs G, Davidian M (2014). The analysis of multivariate longitudinal data: a review. Stat Methods Med Res.

[13] Kassahun-Yimer W, Valle KA, Oshunbade AA, Hall ME, Min YI, Cain-Shields L, Anugu P, Correa A (2020). Joint modelling of longitudinal lipids and time to coronary heart disease in the Jackson Heart Study. BMC Med Res Methodol.

[14] Fieuws S, Verbeke G (2006). Pairwise fitting of mixed models for the joint modeling of multivariate longitudinal profiles. Biometrics.

[15] Fieuws S, Verbeke G, Maes B, Vanrenterghem Y (2008). Predicting renal graft failure using multivariate longitudinal profiles. Biostatistics.

[16] Lubyayi L, Mawa PA, Nabakooza G, Nakibuule M, Tushabe JV, Serubanja J, Aibo D, Akurut H, Tumusiime J, Hasso-Agopsowicz M, Kaleebu P, Levin J, Dockrell HM, Smith S, Webb EL, Elliott AM, Cose S (2020). Maternal latent Mycobacterium tuberculosis does not affect the infant immune response following BCG at birth: an observational longitudinal study in Uganda. Front Immunol.

[17] Soares AP, Kwong Chung CK, Choice T, Hughes EJ, Jacobs G, van Rensburg EJ, Khomba G, de Kock M, Lerumo L, Makhethe L, Maneli MH (2013). Longitudinal changes in CD4+ T-cell memory responses induced by BCG vaccination of newborns. J Infect Dis.

[18] Verbeke G, Molenberghs G. Linear mixed models for longitudinal data. Springer; 2009.

[19] FitzMaurice Garrett M, Laird Nan M, Ware James H. Applied longitudinal analysis. Wiley; 2004.

[20] Weiss RE. Modeling longitudinal data. Springer; 2005.

[21] Fitzmaurice G, Davidian M, Verbeke G, Molenberghs G (2009). Handbooks of modern statistical methods: longitudinal data analysis.

[22] Burton P, Gurrin L, Sly P (1998). Extending the simple linear regression model to account for correlated responses: an introduction to generalized estimating equations and multi-level mixed modelling. Stat Med.

[23] Long J, Ryoo J (2010). Using fractional polynomials to model non-linear trends in longitudinal data. Br J Math Stat Psychol.

[24] Tilling K, Macdonald-Wallis C, Lawlor DA, Hughes RA, Howe LD (2014). Modelling childhood growth using fractional polynomials and linear splines. Ann Nutr Metab.

[25] Royston P, Ambler G, Sauerbrei W (1999). The use of fractional polynomials to model continuous risk variables in epidemiology. Int J Epidemiol.

[26] Benner A (2005). mfp: Multivariable fractional polynomials. R News.

[27] Fieuws S, Verbeke G, Molenberghs G (2007). Random-effects models for multivariate repeated measures. Stat Methods Med Res.

[28] A Sas Macro for Fitting a Multivariate Linear Mixed Model Using the Pairwise Approach. https://ibiostat.be/online-resources/longitudinal#MLMMpw.

