# Supplementary material for: Analysis of multivariate longitudinal immuno-epidemiological data using a pairwise joint modelling approach
Source: BMC Immunol. 2021 Sep 17;22:63. doi: 10.1186/s12865-021-00453-5 (PMC8449434; doi:10.1186/s12865-021-00453-5)
Supplement: Supplementary file 1 — Additional file 1.: Table S1 Number of available samples at each time point for responses to PPD. Table S2 Comparison of Fractional polynomial and conventional higher order polynomials using various fit-statistics. Table S3 Correlation matrix of random effects from the pairwise model. Fig. S1A Observed and fitted mean functions for the cytokine responses to PPD. Fig. S1B Observed and fitted mean functions for the cytokine responses to PPD. [file 12865_2021_453_MOESM1_ESM.docx]

**Table S1. Number of available samples at each time point for responses to PPD**

| **Time point**  **(weeks)** | **LTBI-Negative (n=150)** | **LTBI-Positive (n=132)** | **Total (n=282)** |
| --- | --- | --- | --- |
| **0** | 138 | 116 | 254 |
| **1** | 71 | 58 | 129 |
| **4** | 66 | 53 | 119 |
| **6** | 57 | 56 | 113 |
| **10** | 57 | 51 | 108 |
| **14** | 21 | 32 | 53 |
| **24** | 27 | 29 | 56 |
| **52** | 110 | 95 | 205 |

*Table shows the number of samples assayed at each time point. Half of the infants were expected at time points other than weeks 0 and 52. Weeks 14 and 24 have lower numbers because they were introduced later.*

**Table S2. Comparison of Fractional polynomial and conventional higher order polynomials using various fit-statistics**

| **Outcome** | **Model** | **AIC** | **Deviance** | **R^2^** | **Adjusted R^2^** |
| --- | --- | --- | --- | --- | --- |
| IFN-gamma | Cubic | 2309.9 | 864.83 | 0.2562 | 0.2533 |
|  | FP (0.5, 0.5)* | 2303.5 | 859.99 | 0.2604 | 0.2585 |
|  |  |  |  |  |  |
| TNF | Cubic | 1513.2 | 316.42 | 0.247 | 0.2441 |
|  | FP (0.5, 0.5) | 1508.4 | 315.28 | 0.2498 | 0.2478 |
|  |  |  |  |  |  |
| IL-13 | Cubic | 1976.8 | 567.37 | 0.1463 | 0.143 |
|  | FP (-0.5, 3) | 1961.3 | 557.66 | 0.1609 | 0.1588 |
|  |  |  |  |  |  |
| IL-17A | Cubic | 1527.7 | 318.48 | 0.0544 | 0.0507 |
|  | FP (-2, -2) | 1476.7 | 299.15 | 0.1118 | 0.1095 |
|  |  |  |  |  |  |
| IL-2 | Cubic | 1465.7 | 294.89 | 0.2146 | 0.2115 |
|  | FP (0) | 1457.6 | 293.35 | 0.2187 | 0.2177 |
|  |  |  |  |  |  |
| IL-5 | Cubic | 1844 | 477.81 | 0.1065 | 0.103 |
|  | FP (-1, 3) | 1817.6 | 463.12 | 0.1339 | 0.1317 |
|  |  |  |  |  |  |
| IL-10 | Quadratic | 1400.6 | 271.46 | 0.006027 | 0.003478 |
|  | Linear | 1398.8 | 271.53 | 0.005768 | 0.004495 |

*FP indicates a Fractional Polynomial model with the corresponding powers of time in brackets.

**Table S3. Correlation matrix of random effects from the pairwise model**

|  | **IFN**-**γ** | **IL-10** | **IL-13** | **IL-17** | **IL-2** | **IL-5** | **TNF** |
| --- | --- | --- | --- | --- | --- | --- | --- |
| **IFN-γ** | 1 |  |  |  |  |  |  |
| **IL-10** | **0.81** | 1 |  |  |  |  |  |
| **IL-13** | **0.71** | **0.70** | 1 |  |  |  |  |
| **IL-17** | 0.28 | 0.21 | 0.29 | 1 |  |  |  |
| **IL-2** | 0.21 | -0.01 | 0.14 | 0.09 | 1 |  |  |
| **IL-5** | 0.31 | 0.01 | **0.73** | 0.42 | -0.04 | 1 |  |
| **TNF** | **0.89** | **0.77** | **0.75** | 0.42 | -0.07 | 0.27 | 1 |

Correlation coefficients greater than 0.5 are in bold.

**Figure S1A. Observed and fitted mean functions for the cytokine responses to PPD**


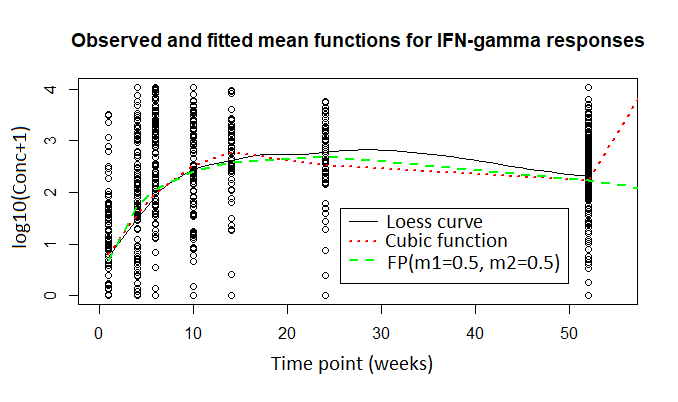

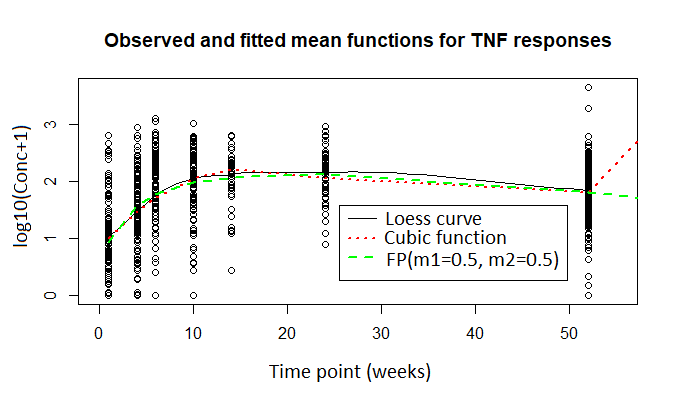

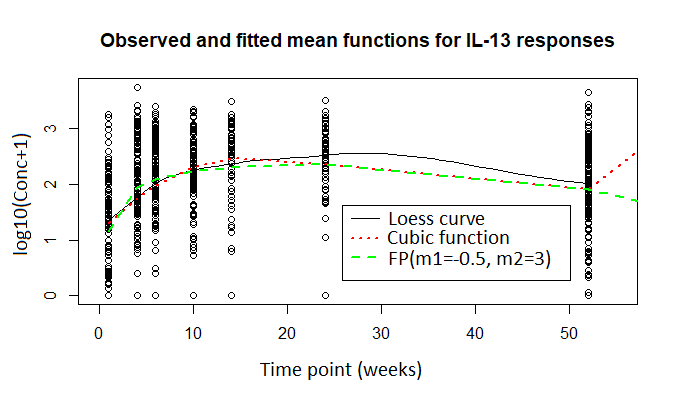

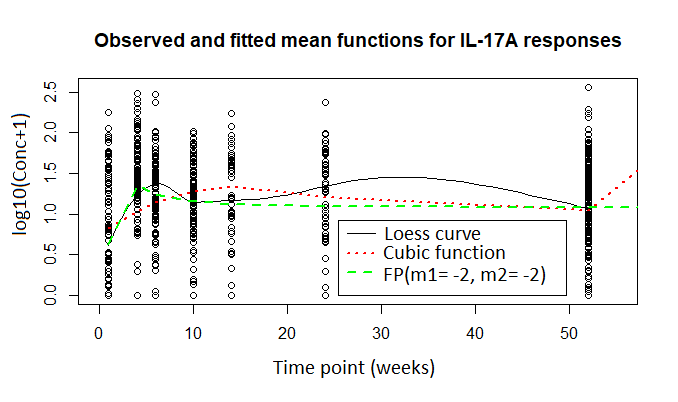


Observed mean functions from loess smoothing are represented by black solid lines, red dotted lines represent the best fitting conventional higher order polynomial function while green dashed lines represent the best fitting fractional polynomial (FP) function of time. The figures in brackets for the FP functions represent the corresponding powers of time.

**Figure S1B. Observed and fitted mean functions for the cytokine responses to PPD**


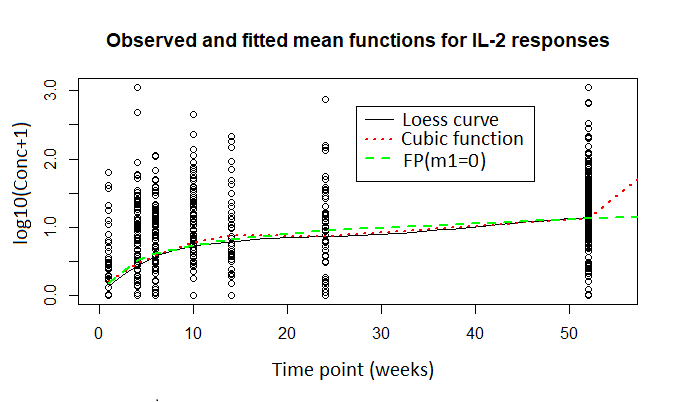

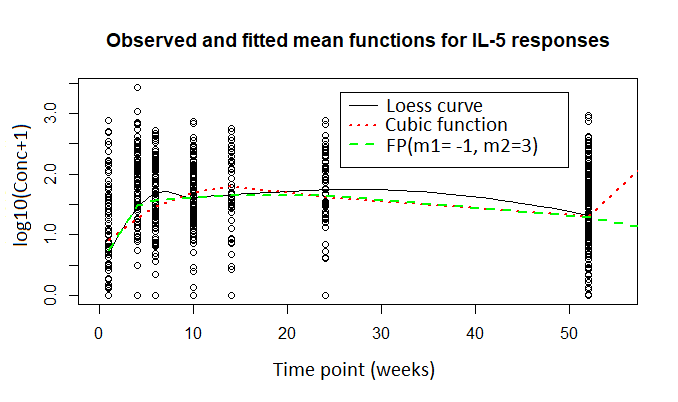

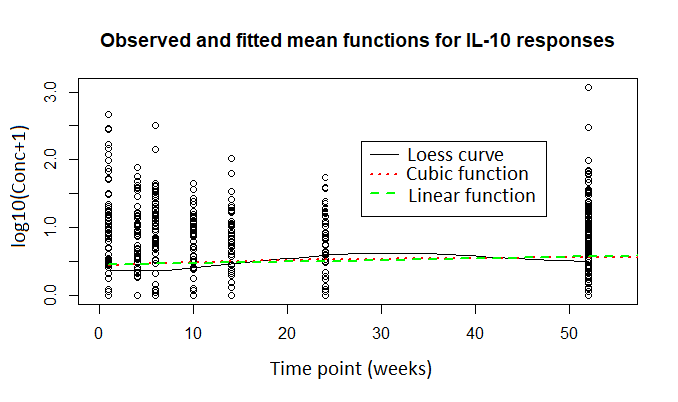


Observed mean functions from loess smoothing are represented by black solid lines, red dotted lines represent the best fitting conventional higher order polynomial function while green dashed lines represent the best fitting fractional polynomial (FP) function of time. The figures in brackets for the FP functions represent the corresponding powers of time.
